# Effect of Silicon on Micronutrient Content in New Potato Tubers

**DOI:** 10.3390/ijms241310578

**Published:** 2023-06-24

**Authors:** Wanda Wadas, Tomasz Kondraciuk

**Affiliations:** Institute of Agriculture and Horticulture, Siedlce University of Natural Sciences and Humanities, B. Prusa 14, 08-110 Siedlce, Poland

**Keywords:** early potatoes, sodium metasilicate, silicon dosage, time of silicon application

## Abstract

Since silicon can improve nutrient uptake in plants, the effect of foliar silicon (sodium metasilicate) application on micronutrient content in early crop potato tuber was investigated. Silicon was applied at dosages of 23.25 g Si∙ha^–1^ or 46.50 g Si∙ha^–1^ (0.25 L∙ha^–1^ or 0.50 L∙ha^–1^ of Optysil) once at the leaf development stage (BBCH 14–16), or at the tuber initiation stage (BBCH 40–1), and twice, at the leaf development and tuber initiation stages. Potatoes were harvested 75 days after planting (the end of June). Foliar-applied silicon reduced the Fe concentration and increased Cu and Mn concentrations in early crop potato tubers under water deficit conditions but did not affect the Zn, B, or Si concentrations. The dosage and time of silicon application slightly affected the Fe and Cu concentration in the tubers. Under drought conditions, the highest Mn content in the tuber was observed when 46.50 g Si∙ha^–1^ was applied at the leaf development stage, whereas under periodic water deficits, it was highest with the application of the same silicon dosage at the tuber initiation stage (BBCH 40–41). The Si content in tubers was negatively correlated with the Fe and B content, and positively correlated with the Cu and Mn content.

## 1. Introduction

Silicon (Si), previously considered a non-essential element for plants, has a potential role in mitigating environmental stresses in plants by regulating physiological, biochemical, and molecular processes [1,2]. It can enhance nutrient uptake and alleviate stress through various mechanisms, such as acting as a physical barrier, improving plant water status (by increasing root water uptake and regulating leaf transpiration), or regulating biochemical cues (such as defence enzyme levels, antioxidant defence, osmolyte regulation, and phytohormone biosynthesis) [1,2,3,4,5]. Silicon can also modulate the expression of genes involved in physiological processes in plants related to mitigating stress [6].

Plant uptake silicon from the soil in the plant-available form of monosilic acid/orthosilicic acid [Si(OH)_4_ or H_4_SiO_4_] [1]. Silicon uptake by the roots may be active (Si uptake is faster than water uptake), passive (Si uptake is similar to water uptake), or rejective (Si uptake is slower than water uptake). The passive mode of silicon uptake is most common among the monocot plants, whereas the rejective mode is the most common among the dicot plants. The different modes of Si uptake are based on molecular mechanisms (channel, pump, carrier) [1,2]. The Si-uptake mechanism of monocot and dicot plants is different due to differences in the root anatomy. Silicon taken up by roots is polymerized to amorthous silica and translocated to the stems and leaves via the transpiration stream through the xylem and deposited as phytoliths or silica bodies in different plant tissues in the apoplast. Different plant species vary in silicon content in tissues. There is also variation in silicon content in different parts of the same plant [2,7]. Plants contain 0.1–10% of silicon on dry weight [2]. Due to the ability to uptake and accumulate silicon, plants are categorozed into three groups: low (<0.2% of Si), intermediate (0.5–1.5% of Si), and high (1.5–10% of Si) Si accumulators. Monocot plants are high or intermediate Si accumulators, and most dicot plants are low Si accumulators [7,8].

A key role in plant Si uptake and transport is played by the aquaporin (AQP) gene family. The specialized Si-transporter genes are *Lsi1*, *Lsi2*, *Lsi3,* and *Lsi6* [7,9,10]. *Solanaceae* plants such as tomatoes and potatoes are poor silicon accumulators, despite having homologs of Si-transporter AQPs. The poor accumulation of silicon by *Solanaceae* is associated with altered spacing between the two conserved asparagine-proline-alanine (NPA) motifs. Spacing between the NPA domains of AQPs is an important feature of Si transporters [9].

Silicon is also applied as foliar spray in the form of silicates, stabilized silicic acid, or silica nanoparticles (Si-NPs). Positive effects of foliar silicon application have been reported both in Si-accumulating and non-accumulating plants. However, the mechanism of Si uptake and translocation in leaf cells is not well understood [7].

Since silicon indicates environmental stress tolerance in plants, the exogenous application of silicon (to soil or foliar) as a low input and environmentally friendly cropping management tool in agriculture has been increasing [1,11,12,13]. In recent years, crop plant growth has been greatly influenced by weather conditions, especially water deficit. Silicon nutrition in crop plants induces drought tolerance [14,15,16,17]. One of the major effects of drought is the disturbed nutrient uptake by plants. The exogenous application of water-soluble forms of Si (to soil or foliar) facilitates the direct uptake of Si by monocot and dicot plants and helps in the uptake of other essential nutrients [4,15,18,19,20].

Although micronutrients are uptaken in small amounts, they play an important role both in plant metabolism and in the human body [21,22,23]. The concentration of micronutrients in plant tissues may be enhanced by biofortification or increasing the bioavailability of micronutrients [3]. Silicon application can alleviate micronutrient deficiency in plants and plant-based food [24]. There are interactions of silicon with essential and beneficial elements in plants, which are plant-specific and dependent on mineral status (deficiency or excess) [2,19]. Regarding the effect of exogenous silicon on the micronutrient content in crop plants, there are some available reports. Under water deficit, silicon fertilization (to soil or foliar) enhanced the Fe, Cu, and Mn uptake by wheat [25], maize [26], rice [27], and sunflower [28], and Fe, Mn, and Zn uptake by melon [29]. Silicon foliar fertilization decreased the Fe, Cu, and Zn content in oat grains [30]. Root application of silicon decreased the Zn and Cu content in cucumber fruit grown in greenhouses [31], whereas foliar silicon application enhanced the content of Fe, Zn, Cu, Mn, and B in cucumber fruit under field conditions [32]. A greenhouse pot experiment in Iran showed that silicon addition to the growth media reduced the Fe, Zn, and Mn content in potato plant, but enhanced the Fe and Zn content in minitubers [33]. Under water deficit, foliar application of silicon enhanced the Fe, Zn, Mn, and Cu content in potato tubers grown in open fields in Egypt and Saudi Arabia [34,35].

Based on the above, it is hypothesized that foliar silicon application could contribute to enhancing micronutrient contents in potato tubers. To test this hypothesis, this study investigated the effect of dosage and time of foliar silicon application on the micronutrient content in early-crop potato tubers.

## 2. Results

Foliar-applied silicon (Si) reduced the Fe content and increased the Cu and Mn content in potato tubers but did not affect the content of Zn, B, and Si (Table 1). Following the application of silicon, the Fe content in tubers was lower, on average, by 2.190 mg∙kg^–1^ DM (over the three-year period), compared with the control treatment without silicon, whereas the Cu content was greater, on average, by 0.295 mg∙kg^–1^ DM, and Mn by 0.554 mg∙kg^–1^ DM.

**Figure 1:**
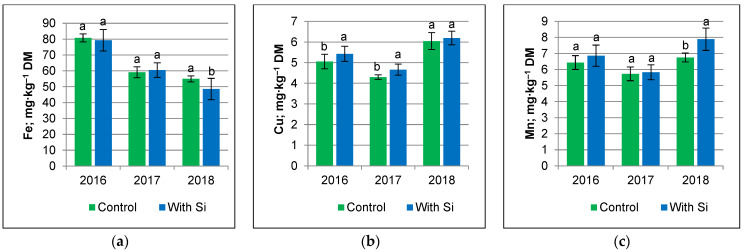
Effect of weather conditions and treatment on Fe (**a**), Cu (**b**), and Mn (**c**) content in potato tuber. Means indicated with the same letters do not differ significantly at *p* ≤ 0.05. Error bars represent SD (*n* = 3 for Control, *n* = 18 for Si treatment).

Foliar silicon application had the greatest effect on the Fe and Mn accumulation by potato tubers in 2018 (very dry and with the lowest content of available Fe and Mn in soil), compared with the Cu accumulation in 2016 (with periodic water deficits) (Figure 1). In 2018, Si-treated tubers had a lower Fe content, on average, by 6.450 mg∙kg^–1^ DM (12%) and greater Mn content, on average by 1.138 mg∙kg^–1^ DM (17%), compared with the untreated control tubers. In 2016, following silicon application, the Cu content in tubers was greater, on average, by 0.372 mg∙kg^–1^ DM (7%).

The dosage and time of silicon application did not significantly affect the micronutrient content in potato tubers except for Mn (Table 2). Following the application of 46.50 g Si∙ha^–1^, the Mn content in tubers was greater, on average, by 0.536 mg∙kg^–1^ DM (8%) (over the three-year period) compared with the dosage of 23.25 g Si∙ha^–1^.

**Figure 2:**
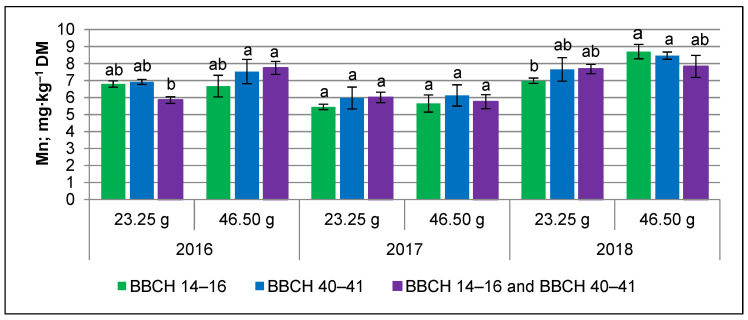
Effect of weather conditions, dosage, and time of silicon application on Mn content in potato tuber. Time of silicon application: BBCH 14–16, leaf development stage; BBCH 40–41, tuber initiation stage; BBCH 14–16 and BBCH 40–41, leaf development and tuber initiation stages. Means indicated with the same letters do not differ significantly at *p* ≤ 0.05. Error bars represent SD (*n* = 3).

The significant effect of the interaction of the year, dosage, and time of silicon application on the Mn content in tubers was found (Figure 2). In 2016, with periodic water deficits, the Mn content in tubers was the greatest following the application of 46.50 g Si∙ha^–1^ at the tuber initiation stage, whereas in the very dry year of 2018, the Mn content in tubers was the greatest following the application of 46.50 g Si∙ha^–1^ at the leaf development stage.

The micronutrient content in tubers depended on the environmental conditions of the potato growth (Table 3). Regardless of treatment (with or without silicon), most of the Fe, Zn, and B—and at the same time, the least of the Si—were accumulated by potato tubers in 2016, with periodic water deficits during tuber bulking and with the highest content of available Fe, Zn, and B in soil. Most of the Cu, Mn, and Si were accumulated in tubers in the very dry growing season of 2018, with the lowest content of available Cu and Mn in soil and with an acid soil reaction.

The Si content in tubers was negatively correlated with Fe and B content, and positively correlated with Cu and Mn content (Table 4). Significant positive correlations were also observed between Fe content and content of Zn and B, the content of Zn and B, and the content of Cu and Mn.

Silicon application increased the early potato yield by an average of 2.43 t∙ha^–1^ (over the three-year period), but did not affect average tuber weight and dry matter content in tubers (Table 5). The tuber yield and average tuber weight were negatively correlated with the Cu, Mn, and Si content in tubers (Table 6). The tuber yield was positively correlated with the Fe and B content in tubers, whereas the dry matter content in tubers was positively correlated with the Cu, Mn, and Si content.

## 3. Discussion

The recent progress of research on plant nutrition suggests that the use of some trace elements, such as iodine (I), selenium (Se), silicon (Si), titanium (Ti), or vanadium (V), can improve plant nutrient use efficiency and abiotic stress tolerance [36,37]. Silicon fertilization in sustainable crop production has gained more attention [12,13,20,38]. Studies showed the regulatory role of silicon in mitigating plant nutritional stresses [18,19]. Silicon can partially alleviate micronutrient deficiency in plants [24]. In the present study, foliar silicon (sodium metasilicate, Na_2_SiO_3_) application reduced the Fe accumulation and increased the Cu and Mn accumulation by tubers of very early potato cultivar ‘Catania’ grown on sandy loam soil, especially under water deficit during tuber bulking. The exogenous silicon did not affect the Zn, B, and Si content in tubers. In potatoes, the direct uptake of nutrients into the developing tubers across the periderm is also possible. In mature tubers, this process will be limited due to the suberization of the periderm [39]. Micronutrients are taken up from soil via passive diffusion across the plasma membrane [40]. The mechanisms of Si-induced alterations in plants under mineral nutrient stress are unclear. The possible molecular-level mechanisms of Si-induced micronutrient uptake by plants under drought stress include improving cell membrane stability by reducing oxidative damage and stimulating H^+^-ATPase activity, as well as regulating the expresion of ion transporter genes. Silicon application decreases oxidative damage in plants by increasing the activity of antioxidant enzymes such as catalase (CAT), peroxidase (POD), superoxide dismutase (SOD), glutathione reductase (GR), and ascorbate peroxidase (APX) [11,14,15,17,19]. Potatoes are poor silicon accumulators [9,41]. Silicon taken up by roots is translocated to the stems and leaves via the transpitation stream through the xylem. The mechanism of the Si uptake and translocation in leaf cell is not well understood [7]. The soil or foliar application of sodium metasilicate differently affect Si concentration in potato plants. Soil application of silicon provided highest Si concentration in stems, whereas the foliar silicon application provided highest Si concentration in leaves. Neither soil or foliar silicon application had an effect on the Si concentration in tubers [42], which was confirmed in the present study.

The micronutrient content in potato tubers depends on the genotype, maturity stage, and growth conditions [43,44,45,46]. Previous studies showed that foliar-applied potassium silicate (K_2_Si_2_O_2_) increased the Fe, Zn, Cu, and Mn in tubers of the medium-early potato cultivar ‘Diamond’ grown in newly reclaimed sandy soil in Egypt [34]. The foliar-applied silicon dioxide (SiO_2_) nanoparticles increased the Fe and Zn content in tubers of medium-early cultivar ‘Hermes’ grown in Saudi Arabia [35]. In the present study, the foliar-applied sodium metasilicate (Na_2_SiO_3_) reduced the Fe content and did not affect the Zn content in tubers of very early potato cultivar ‘Catania’. The potatoes were grown in the soil with a low–medium content of available Fe and B. In B-deficient conditions, silicon can reduce the Fe concentration in roots and increase the Fe concentration in leaves, increasing its mobility [20]. The effect of silicon on the Fe nutrition of different crop plants in low, optimal, and high Fe supply conditions was clearly demonstrated. In general, silicon alleviates Fe deficiency. It affects Fe availability in the rhizosphere and the root apoplast and the expression of genes involved in Fe transport, thus influencing Fe uptake, translocation, and distribution within different plant organs and tissues. The Si-alleviating effect on Fe plant deficiency is species specific and pH dependent [19]. In nutrient or soil solution, all forms of solid silica are dissolved to a limited extend in water around pH 7.0. The nutrient solution pH used to study Fe and other micronutrients deficiency is normal around 7.5–8.0, and to study metal toxicity, 5.0–6.5 [24]. Recent studies have reported that the addition of Si to the growth media may cause the formation of Fe plaque at the root surface and an apoplastic obstruction, thus decreasing Fe uptake even at optimal Fe supply [8]. In the present study, the effect of silicon was evaluated in the presence of iron chelate. The Si-based biostimulant Optysil used in the study is a mixture of sodium metasilicate and iron chelate (Fe-EDTA), which is difficult to decriminate the single effect of silicon and iron on plants. Nutrient foliar absorption depends on the age of the leaf and environmental factors (temperature, air humidity). The young leaves absorb more nutrients than the older ones [47]. The Fe concentration in potato tubers is affected by the growing location [48]. The sodium and potassium silicate applied to soil or leaves increased the superoxide dismutase (SOD) activity and manganese use efficiency [18,34], which was confirmed in the present study. It is reported that silicon application enhances the accumulation of macronutrients and Mn, which decreases stress arising from ionic damage and increases drought tolerance and improves plant growth [11,19,49]. In potatoes, sodium metasilicate application (to soil or foliar) has different effects on the leaf, stem, and tuber Si concentration. Both soil and foliar silicon application did not affect the Si concentration in tubers [20], which was confirmed in the present study.

Among the same crop plants, the genotype-dependent response to silicon nutrition may be observed. Moreover, the dosage and the time of plant stage-dependent application of silicon are important [18]. In general, foliar silicon application is practical only at a very low dosage and starting in early plant-stage development [50,51]. There is scarce knowledge of the effect of different dosages and times of silicon application on the micronutrient concentration in potato tubers. In the present study, both the dosage and the time of silicon application did not significantly affect the micronutrient content in early-harvested potatoes (75 days after planting), except for Mn. Silicon at the dosage of 46.50 g Si∙ha^–1^ stimulated the Mn accumulation more by tubers than at 23.25 g Si∙ha^–1^. The effect of silicon application depended on the weather conditions during potato growth. Under drought stress (2018 season), the Mn accumulation by tubers was most stimulated by silicon application at 46.50 g Si∙ha^–1^ (0.50 L∙ha^–1^ of Optysil) at the leaf development stage (BBCH 14–16), whereas under periodic water deficits during tuber bulking (2016 season) by application, the same silicon dosage at the tuber initiation stage (BBCH 40–41). In a previous study in Egypt, potassium silicate (K_2_Si_2_O_2_) at 2 mL∙L^–1^ was applied 3 times with 10-day intervals starting at 40 days after the planting date [34]. In Saudi Arabia, silicon dioxide (SiO_2)_ nanoparticles (SiO_2_-NPs) at 25 mg∙L^–1^ and 50 mg∙L^–1^ were applied twice, at 45 and 65 days after planting (DAP). The Fe and Zn content in potato tubers grown under water deficit conditions was higher following an application of 50 mg∙L^–1^ SiO_2_-NPs [35]. The effect of foliar silicon application depends on the pH of water used to prepare the working solution. In water around pH 7.0, the silicon concentration is less than 2 mM, or above this, polycondensation takes place and colloidal particles could be formed. In the nutrient solution pH above 7.5–8.0, silicon concentrations below 2 mM can be tested [24].

A relationship between silicon deposition in plant tissue and the content of essential and beneficial elements was reported in previous studies [2,19], and this relationship was confirmed in the present study, which found a negative correlation between the Si content in immature potato tubers and the Fe and B content, while a positive correlation was found between the Si content and the Cu and Mn content.

A relationship was also reported between yield and mineral concentrations in potato tubers. It has been observed that higher-yielding cultivars sometimes have lower mineral concentrations in tubers than lower-yielding cultivars grown in the same location [44]. ‘Catania’ is a high-yielding very early potato cultivar [52]. In the present study, the foliar-applied silicon (Na_2_SiO_3_) increased yield and average tuber weight [50], but did not affect the content of dry matter in tubers [53]. The yield and average tuber weight were negatively correlated with the Cu, Mn, and Si content in tubers, but the dry matter content in the tubers was positively correlated with the content of these micronutrients.

As mentioned, the micronutrient content in potato tubers depends on the genotype, maturity stage, and growth conditions [43,44,45,46]. The most important factors affecting the micronutrient uptake by plants are their levels in the soil, soil moisture, and pH. The bioavailability of micronutrient present in a soil solution as cations (e.t. Fe, Zn, B) increases with increasing soil acidity [40,54], which was confirmed in the present study.

## 4. Materials and Methods

### 4.1. Plant Material, Experimental Design, and Treatment

The study material included tubers of the very early potato cultivar ‘Catania’ obtained from a 3-year field experiment conducted at the Siedlce University of Natural Sciences and Humanities experimental field (52°03′ N, 22°33′ E, 160 m above sea level) in 2016–2018. ‘Catania’ is one of the most widely grown early crop potato cultivars in central-eastern Poland, with medium–high soil and water requirements [52].

The field experiment was located on Haplic Luvisol (LV-ha) with a sandy loam texture, with low organic matter content and an acidic–slightly acidic reaction. The soil was characterized by a high content of available phosphorus, medium–high content of potassium and low–medium content of magnesium, and a low–medium content of available B, Cu, and Fe, and a medium content of Mn and Zn (Table 7). The soil chemical properties were determined by standard methods in the laboratory of the National Chemical and Agricultural Station in Warsaw (Accreditation Certificate No. AB 312, Polish Center for Accreditation, Warsaw): organic matter with Turin’s method [55]; pH with a potentiometric method in 1 M KCl solution [56]; available phosphorus (P) with a spectrophotometric method [57]; potassium (K) with the flame atomic emission spectroscopy (FAES) method [58] and magnesium (Mg), zinc (Zn), manganese (Mn), iron (Fe), and copper (Cu) with the flame atomic absorption spectroscopy (FAAS) method [59,60,61,62,63].

Air temperature and rainfall in the potato growing period were recorded at the Siedlce University of Natural Sciences and Humanities Meteorological Station. The growing season of 2016 was warm, with periodic water deficits. The following growing season (2017) was warm and moderately wet, whereas the growing season of 2018 was warm and very dry (Table 8).

The effect of foliar silicon (Si) application using the commercial product Optysil (Intermag Ltd., Olkusz, Poland) on the micronutrient content in new potato tubers was investigated. Optysil contains 93 g Si (7.8 m/m) in the form of sodium metasilicate (Na_2_SiO_3_) and 24 g Fe (2 m/m) in the form of iron chelate (Fe-EDTA) per liter. The recommended dosage of the Optysil for potato harvested at fully ripe is 0.50 L∙ha^–1^ (46.50 g Si∙ha^–1^) at the tuber initiation stage and repeating the treatment at tubers reaches 70–80% of the final mass, with the option at the beginning of plant growth. The growth period of early crop potatoes is short, such as that in the present study whivh uses two dosages of silicon—23.25 g Si∙ha^–1^ and 46.50 g Si∙ha^–1^ (0.25 L∙ha^–1^ and 0.50 L∙ha^–1^ of Optysil).

The field experiment was established as a two-factor split-plot design with a control object, with three replications. The main plots were silicon dosage—23.25 g Si∙ha^–1^ and 46.50 g Si∙ha^–1^ (0.25 L∙ha^–1^ and 0.50 L∙ha^–1^ of Optysil), and the subplot times of silicon application—once at the leaf development stage (BBCH 14–16 stage) or tuber initiation stage (BBCH 40–41), and twice at the leaf development and tuber initiation stages (BBCH 14–16 and BBCH 40–41). Potato plants sprayed with water were used as a control (without Si). A single control plot was located between the main plots in each replication. The plot area was 16.2 m^2^ (96 plants per plot).

Potato cultivation was carried out according to common agronomical practices described in a previous paper [50]. Farmyard manure was applied in autumn at a rate of 25 t∙ha^–1^, and mineral fertilizers were applied at rates of 80 kg N (ammonium nitrate), 35 kg P (superphosphate), and 100 kg K (potassium sulfate) per hectare in spring. In total, 6-week pre-sprouted seed potatoes were planted in the first decade of April, with in-row spacing of 0.25 m and 0.675 m between rows. Potatoes were harvested at the end of June (75 days after planting). The tuber yield and tuber yield components were determined [50].

### 4.2. Tuber Sampling and Laboratory Analyses

For laboratory studies, 50 different-sized tubers (with diameters of 30–60 mm) were taken from 10 successive randomized plants per plot. Tuber sections cut out along the longitudinal axis from the tuber tip to the stolon attachment site were crumbled and 2-stage dried at an initial temperature of 60 °C, and then, at 105 °C to determine the tuber dry matter with the gravimetric method [64]. The contents of Fe, Zn, Cu, B, Mn, and Si were determined with the inductively coupled plasma optical emission spectrometry (ICP-OES) method (Optima 8300, Perkin Elmer, Waltham, MA, USA) after sample mineralization in a mixture of concentrated HNO_3_ and 20% H_2_O_2_ (3:1) in a microwave system (Ethos Plus, Milestone, Sorisole, Italy) [65]. All assays were performed in duplicate, and the mean contents of micronutrients were expressed as milligrams per kilogram of potato tuber dry matter (DM) ± standard deviation (SD).

### 4.3. Data Analysis

The results of the study were subjected to a two-way analysis of variance (ANOVA) for split-plot design (silicon dosage × time of silicon application) with a control object during three years. The orthogonal contrast was used to compare the silicon treatments with the control. The significance of differences between the compared means has been established using Tukey’s HSD (Honestly Significant Difference) test (*p* ≤ 0.05).

The relationships between the micronutrient content in tubers and the relationships between the micronutrient content and the yield and average tuber weight, and the dry matter content in tuber were determined by calculating Pearson’s linear correlation coefficients (*n* = 21).

## 5. Conclusions

Foliar-applied silicon (sodium metasilicate) reduced the Fe concentration and increased Cu and Mn concentration in early crop potato tubers under water deficit conditions, but did not affect the Zn, B, and Si concentrations. The dosage and time of silicon application did not significantly affect the Fe and Cu concentration in the tubers. Under drought conditions, the Mn concentration in the tubers was the highest with the application of 46.50 g Si∙ha^–1^ at the leaf development stage, whereas under periodic water deficiency it was highest with the application of the same silicon dosage at the tuber initiation stage. The Si content in tubers was negatively correlated with the Fe and B contents and positively correlated with the Cu and Mn contents. These results increased current knowledge on the effect of foliar-applied silicon on micronutrient concentration in potato tubers. The growth period of the potatoes was short, only 75 days from planting. Thus, future studies are necessary to evaluate the responses of different potato cultivars to exogenous silicon and determine the effect of different silicon sources and concentrations on nutrient accumulation by potato tubers.

## Figures and Tables

**Table 1:** Effect of silicon (Si) on micronutrient content in tubers; mg∙kg^–1^ DM.

Treatment	Fe	Zn	Cu	Mn	B	Si
Control	64.94 ± 12.22 a	15.10 ± 1.18 a	5.131 ± 0.806 b	6.304 ± 0.562 b	5.531 ± 1.506 a	0.228 ± 0.122 a
With Si	62.75 ± 14.11 b	15.25 ± 1.48 a	5.426 ± 0.708 a	6.858 ± 1.042 a	5.534 ± 1.381 a	0.250 ± 0.124 a

Means indicated with the same letters do not differ significantly at *p* ≤ 0.05. Values are mean of 3 years ± SD (*n* = 3 for Control, *n* = 18 for Si treatment).

**Table 2:** Effect of dosage and time of silicon (Si) application on micronutrient content in tubers; mg∙kg^–1^ DM.

Dosage and Time of Si Application	Fe	Zn	Cu	Mn	B	Si
Silicon dosage; g Si∙ha^–1^						
23.25 g	61.56 ± 13.61 a	15.11 ± 1.45 a	5.412 ± 0.670 a	6.590 ± 0.830 b	5.445 ± 1.294 a	0.253 ± 0.126 a
46.50 g	63.94 ± 14.76 a	15.40 ± 1.54 a	5.440 ± 0.756 a	7.126 ± 1.172 a	5.624 ± 1.482 a	0.249 ± 0.124 a
Time of silicon application ^1^						
BBCH 14–16	63.16 ± 13.56 a	15.49 ± 1.61 a	5.468 ± 1.358 a	6.710 ± 1.091 a	5.434 ± 1.346 a	0.246 ± 0.123 a
BBCH 40–41	64.79 ± 16.45 a	15.34 ± 1.54 a	5.387 ± 0.739 a	7.111 ± 2.243 a	5.604 ± 1.710 a	0.260 ± 0.132 a
BBCH 14–16 and BBCH 40–41	60.30 ± 12.46 a	14.93 ± 1.34 a	5.423 ± 0.734 a	6.755 ± 1.093 a	5.565 ± 1.558 a	0.249 ± 0.124 a

^1^ BBCH 14–16, leaf development stage; BBCH 40–41, tuber initiation stage; BBCH 14–16 and BBCH 40–41, leaf development and tuber initiation stages. Means indicated with the same letters do not differ significantly at *p* ≤ 0.05. Values are mean of 3 years ± SD (*n* = 9 for Si dosage, *n*= 6 for Time of Si application).

**Table 3:** Effect of weather conditions on micronutrient content in tubers; mg∙kg^–1^ DM.

Years	Fe	Zn	Cu	Mn	B	Si
2016	79.47 ± 6.30 a	16.90 ± 0.62 a	5.373 ± 0.296 b	6.801 ± 0.643 b	7.275 ± 0.457 a	0.153 ± 0.075 c
2017	60.26 ± 4.41 b	14.16 ± 0.51 b	4.607 ± 0.282 c	5.812 ± 0.450 c	5.273 ± 0.305 b	0.177 ± 0.017 b
2018	49.44 ± 6.66 c	14.64 ± 0.73 b	6.171 ± 0.333 a	7.724 ± 0.763 a	4.054 ± 0.321 c	0.412 ± 0.035 a

Means indicated with the same letters do not differ significantly at *p* ≤ 0.05. Values are mean of treatments ± SD (*n* = 21).

**Table 4:** Pearson correlation coefficients between micronutrient content in potato tuber (*n* = 21).

Specification	Fe	Zn	Cu	Mn	B	Si
Fe	1					
Zn	0.7947 **	1				
Cu	−0.3335	0.1640	1			
Mn	−0.2287	0.2394	0.8706 **	1		
B	0.9293 **	0.7844 **	−0.3519	−0.2594	1	
Si	−0.7476 **	−0.3877	0.8089 **	0.7087 **	−0.8033 **	1

** Significant at *p* ≤ 0.01.

**Table 5:** Effect of Silicon (Si) on tuber yield, average tuber weight and dry matter content in tubers.

Treatment	Tuber Yield; t∙ha^–1^	Tuber Weight; g	Dry Matter; %
Control	18.55 ± 6.65 b	29.6 ± 6.8 a	18.65 ± 2.92 a
With Si	20.98 ± 7.09 a	30.0 ± 7.0 a	18.82 ± 2.70 a

Means indicated with the same letters do not differ significantly at *p* ≤ 0.05. Values are mean of 3 years ± SD (*n* = 3 for Control, *n* = 18 for Si treatment).

**Table 6:** Pearson correlation coefficients between tuber yield, average tuber weight and dry matter content in tuber, and micronutrient content (*n* = 21).

Specification	Fe	Zn	Cu	Mn	B	Si
Tuber yield	0.5451 *	0.1509	−0.8129 **	−0.7200 **	0.5515 **	−0.8618 **
Tuber weight	−0.0983	−0.4246	−0.5386 *	−0.5422 *	−0.1387	−0.2982
Dry matter	−0.6022	−0.1501	0.9220 **	0.7873 **	−0.6475 **	0.9497 **

* Significant at *p* ≤ 0.05, ** significant at *p* ≤ 0.01.

**Table 7:** Soil chemical properties on the layer of 0–20 cm at the experimental site.

Years	Organic Matter; %	Soil pH_KCl_	Available Nutrients; mg∙kg^–1^							
			P	K	Mg	B	Mn	Cu	Zn	Fe
2016	1.49	5.5	102	95	42	0.45	98.2	4.9	8.2	719
2017	1.59	5.7	114	124	35	0.29	90.3	5.2	7.2	665
2018	1.34	5.2	97	93	23	0.38	74.7	2.7	6.3	610

**Table 8:** Mean air temperature and precipitation total in the potato growing period.

Years	Temperature; °C			Rainfall; mm		
	April	May	June	April	May	June
2016	9.1	15.1	18.4	28.7	54.8	36.9
2017	6.9	13.9	17.8	59.6	49.5	57.9
2018	13.1	17.0	18.3	34.5	27.3	31.5
Many year (1981–2010)	8.3	12.2	16.8	41.2	53.0	63.8

## Data Availability

Not applicable.
